# Spontaneous regression of non-small cell lung cancer after biopsy of a mediastinal lymph node metastasis: a case report

**DOI:** 10.1186/s13256-015-0702-9

**Published:** 2015-09-17

**Authors:** Alberto Lopez-Pastorini, Till Plönes, Michael Brockmann, Corinna Ludwig, Frank Beckers, Erich Stoelben

**Affiliations:** 1 Department of Thoracic Surgery, University Medical Center Witten/Herdecke, Lung Clinic Merheim, Campus Cologne, Ostmerheimerstr. 200, 51109 Cologne, Germany; 2 Department of Pathology, University Medical Center Witten/Herdecke, Clinic Merheim, Campus Cologne, Ostmerheimerstr. 200, 51109 Cologne, Germany

**Keywords:** Spontaneous regression, Immunologic reaction, Non-small cell lung cancer, Biopsy, Complete remission

## Abstract

**Introduction:**

Spontaneous regression of cancer is defined as a complete or partial, temporary or permanent disappearance of tumor in the absence of specific therapy. With only a few cases reported, spontaneous regression is extremely rare in primary lung cancer. Regarding spontaneous regression in lung cancer, recent investigations revealed the role of immunological mechanisms, thus indicating potential treatment options by specific immunotherapy in the future.

**Case presentation:**

A 76-year-old Caucasian man with progressive dyspnea presented to our hospital. A computed tomography scan revealed a tumor mass in the upper lobe of his right lung and enlarged mediastinal lymph nodes. A biopsy of a paratracheal lymph node by mediastinoscopy disclosed metastatic lung cancer. By immunohistochemical findings the tumor was classified as large cell carcinoma. Diagnosed with clinical stage IIIA non-small cell lung cancer, a neoadjuvant therapy concept was indicated. However, before starting chemoradiation, a computed tomography scan showed a regression of both the tumor mass in the upper lobe of his right lung and the mediastinal lymphadenopathy. As a repeated computed tomography scan showed further regression, we agreed with our patient to perform routine follow-up instead of starting therapy. To date, no relapse has been reported.

**Conclusions:**

Given the circumstances that regression started after the biopsy and involved both the tumor in the upper lobe of his right lung and the mediastinal lymph node metastases, an immune response is a reasonable explanation for the observed spontaneous regression in this case.

## Introduction

Spontaneous regression (SR) of cancer is an unusual event and extremely rare in primary lung cancer. It is defined as a complete or partial, temporary or permanent disappearance of tumor in the absence of anticancer therapy. Although the concrete mechanisms of SR remain unknown, recent investigations revealed the role of immunological mechanisms involved in SR of lung cancer.

Here we report the case of a patient with an advanced-stage non-small cell lung cancer (NSCLC) that completely regressed after a biopsy of a mediastinal lymph node metastasis.

## Case presentation

**Fig. 1:**
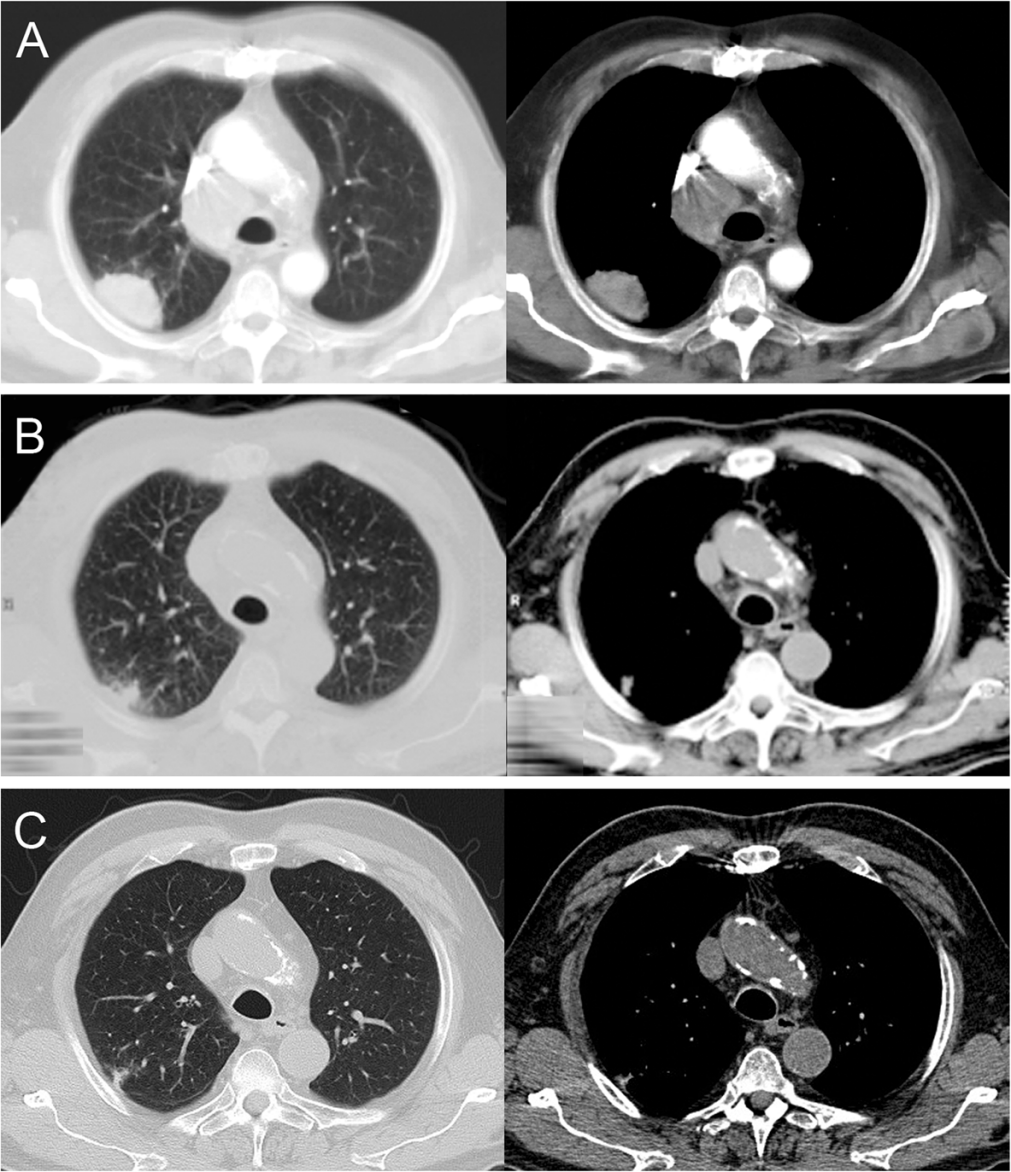
**a** Initial chest computed tomography scan showing the tumor in the upper lobe of the right lung and enlarged mediastinal lymph nodes in the paratracheal position. **b** Computed tomography scan after 2 months, **c** after 1 year

**Fig. 2:**
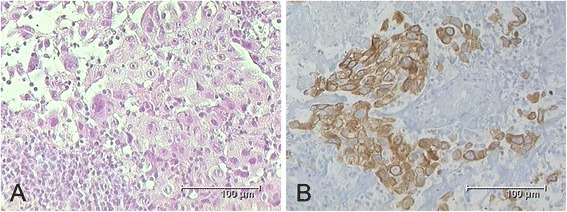
**a** Tissue obtained by biopsy of a right paratracheal lymph node showing metastatic cells of large cell carcinoma. **b** Immunohistochemical staining showing cytokeratin positivity (KL-1) of tumorous cells within the lymphatic tissue

A 76-year-old Caucasian man with progressive dyspnea for the last two months was admitted to our hospital. A contrast-enhanced computed tomography (CT) scan of his chest disclosed an oval-shaped tumor mass in the upper lobe of his right lung, adjoining the pleura and 6 × 5 × 3cm in size (Fig. 1a). In addition, the CT scan showed enlarged mediastinal lymph nodes in the right paratracheal position. Our patient was an active smoker with a cumulative exposure of 50 pack-years and had a medical history of hypertension and hyperlipidemia. His actual medication consisted of an ACE inhibitor and a statin with no changes for the last 2 years. Physical and laboratory examinations revealed no abnormal findings. A mediastinoscopic biopsy of the mediastinal lymph nodes revealed metastatic cells of a poorly differentiated NSCLC in a paratracheal lymph node (4R) (Fig. 2). Immunohistochemical findings showed positive staining for cytokeratin (CK) 7 but no reactivity with antibodies against TTF1, CK5/6, p63 and napsin. Furthermore the cells were negative for CD56, chromogranin and synaptophysin. By these findings, the tumor was classified as large cell carcinoma. Further staging procedures including bone scintigraphy, abdominal CT and head magnetic resonance imaging (MRI) disclosed no distant metastases, so that our patient was diagnosed with clinical stage IIIA (T2bN2M0) NSCLC. Consequently, a neoadjuvant concept consisting of a combined chemoradiation was indicated.

**Fig. 3:**
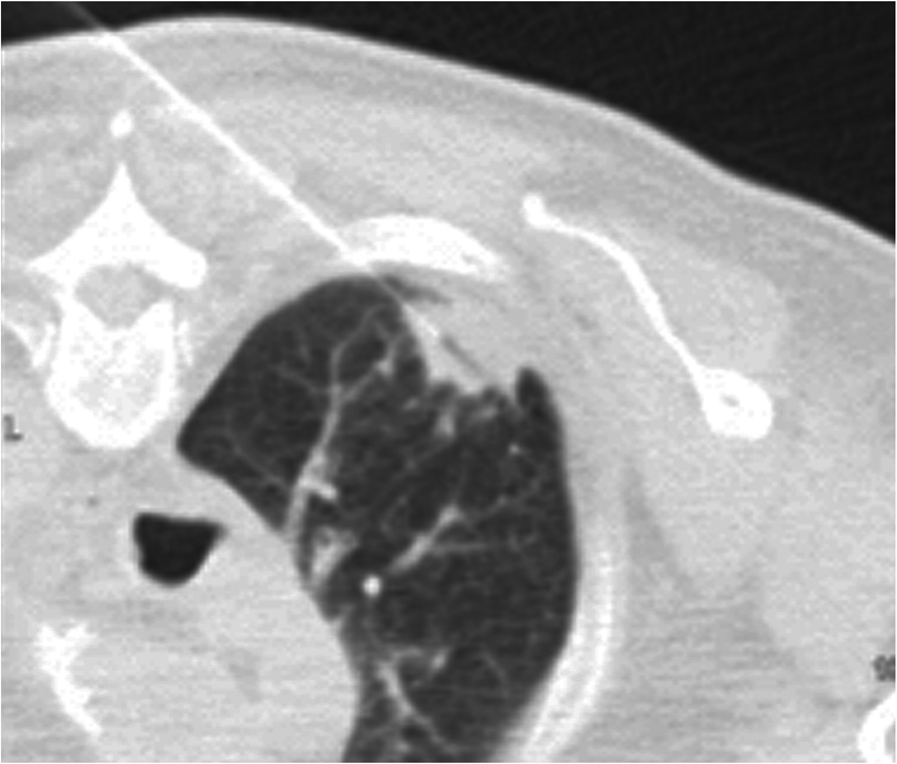
Computed tomography-guided fine-needle biopsy of the tumor in the upper lobe of the right lung

However, CT planning before starting therapy and 2 weeks after mediastinoscopy showed a decrease of both the tumor mass in the upper lobe of his right lung and the mediastinal lymph nodes. We conducted a CT-guided fine-needle biopsy of the tumor in the upper lobe of his right lung (Fig. 3). The histological examination showed extended necrosis but failed to prove malignant cells. In addition, the tissue obtained by mediastinoscopy was examined by a second pathologist, who confirmed the prior diagnosis of NSCLC. A repeated chest CT scan showed further regression of the tumor and the mediastinal lymphadenopathy (Fig. 1b). At this time we agreed with our patient to postpone chemoradiation and instead perform a routine follow-up by an annual chest CT scan. After 1 year, a CT scan revealed the almost complete disappearance of the tumor in the upper lobe of his right lung and a decrease of the mediastinal lymph nodes to normal size (Fig. 1c). Up to the present day, our patient has received no anticancer therapy. He remains in follow-up care in our hospital and after 7 years no relapse has been reported.

## Discussion

The most commonly accepted criteria for spontaneous regression (SR) of cancer were postulated by Everson and Cole in 1959. Here SR is defined as the partial or complete, temporary or permanent, disappearance of the tumor in the absence of any specific therapy [1]. The actual incidence of spontaneous regression is unknown [2]. By the Everson and Cole definition, it was estimated to be not more than 1 in 60,000 to 100,000 cases [3].

Compared with other solid tumors in which SR is more frequently reported, like renal cell carcinoma or malignant melanoma, SR of primary lung cancer is extremely rare [2, 4]. Kumar and colleagues list only two cases of primary lung cancer from 1951 to 2008 that meet the definition of Everson and Cole [5].

There are several suggested mechanisms including immunological factors, hormonal changes, trauma or variations in blood supply [2, 4, 6].

Once regarded as a poorly immunogenic tumor, NSCLC has recently emerged as a target for promising cancer vaccines and immune modulators [7, 8]. Nivolumab, a monoclonal antibody for the human programmed cell death 1 (PD-1) receptor, has been approved this year in the United States for the treatment of squamous NSCLC. In a phase I clinical trial of nivolumab in 2012 33% of the squamous cell lung cancers and 12% of the nonsquamous cell tumors responded with an overall median duration of response of 74 weeks [7]. Interestingly, a smoking history has been associated with an improved response to PD-1 blockade due to a higher neoantigen burden in smokers [9].

Referring to lung cancer, recent articles describe changes in the immunological environment of the tumor that can affect both oncogenesis and regression. Scheider *et al*. showed an accumulation of regulatory T cells in pulmonary adenocarcinoma and metastatic lymph nodes, resulting in a local decrease of antitumor immune response by natural killer cells [10]. Iwakami and colleagues reported an infiltration of CD8-positive lymphocytes in small cell lung cancer that regressed spontaneously, indicating that T cell-mediated cytotoxity is a possible mechanism of SR in lung cancer [11]. Isobe *et al*. demonstrated an integrated immune response consisting of immunoglobulin G (IgG) antibodies, CD4 and CD8 T cells against a NY-ESO-1-expressing NSCLC experiencing SR [12].

Several cases of SR have implicated surgery or biopsy conducted on the primary tumor or the metastases as elements that can induce an immunological response [13]. Cole reported that 71 out of 176 cases of SR in cancer were associated with some type of operative trauma [3, 14]. This may be relevant to our patient, who received a biopsy of metastatic lymph nodes before SR was noted. The biopsies were performed with endoscopic forceps, this causing a disruption of the lymph nodes, potentially leading to a release of antigens with activation of the immune system.

There are two important limitations in our case that need to be discussed. In the first place, we were not able to prove malignancy of the tumor in the upper lobe of the right lung. Histological examinations showed extensive necrosis, potentially caused by tumor disintegration. However, being a peripheral lesion, also a lung infarction, an organizing pneumonia or a limited pulmonary vasculitis should also be considered as possible cause. Immunohistochemical analyses to distinguish tumor necrosis from such an infarction, for example by reticulin or keratin staining, were not performed. In conclusion, there is no certain evidence of a primary tumor in this case. Second, by microscopic and immunohistochemical findings, the tumor cells in the paratracheal lymph node were classified as large cell carcinoma. This is rather a diagnosis of exclusion and does not prove a malignancy of the lung exclusively. Carcinomas of several origins and entities show CK7 positivity, so that in theory, this could be a metastasis from elsewhere in combination with a lung infarction.

Summing up our findings, we had a highly suspicious pulmonary lesion with a proven metastatic lymph node in an expectable position for lung cancer. In addition, neither did the staging procedures reveal any other lesions nor did a malignancy develop elsewhere in 7 years of follow-up. Therefore, in our opinion, the most reasonable explanation for the findings in our case is a primary lung cancer with lymph node metastases.

## Conclusions

The present case complies with the above mentioned criteria of SR. In our case, cells of a large cell carcinoma were proven by pathological findings in a mediastinal lymph node metastasis. Although our patient received no treatment, a near complete remission could be observed within 1 year. Given the circumstances that the regression started after the biopsy and involved both the tumor in the upper lobe of his right lung and the mediastinal lymph node metastases, an immune response is a reasonable explanation for the observed SR in this case.

## Consent

Written informed consent was obtained from the patient for publication of this case report and any accompanying images. A copy of the written consent is available for review by the Editor-in-Chief of this journal.

## Abbreviations

CT: computed tomography
CK: cytokeratin
IgG: immunoglobulin G
MRI: magnetic resonance imaging
NSCLC: non-small cell lung cancer
PD-1: human programmed cell death 1
SR: spontaneous regression
